# Research on Indoor Environment Prediction of Pig House Based on OTDBO–TCN–GRU Algorithm

**DOI:** 10.3390/ani14060863

**Published:** 2024-03-11

**Authors:** Zhaodong Guo, Zhe Yin, Yangcheng Lyu, Yuzhi Wang, Sen Chen, Yaoyu Li, Wuping Zhang, Pengfei Gao

**Affiliations:** 1 College of Software, Shanxi Agricultural University, Jinzhong 030801, China; g1307256189@126.com (Z.G.); yinz9949@126.com (Z.Y.); l1978453400@163.com (Y.L.); wyzn98@163.com (Y.W.); czuxxs@163.com (S.C.); 2 College of Agricultural Engineering, Shanxi Agricultural University, Jinzhong 030801, China; liyaoyu1998@126.com; 3 College of Animal Science, Shanxi Agricultural University, Jinzhong 030801, China; gpf800411@126.com

**Keywords:** pig barn, dung beetle optimization algorithm, TCN, GRU, environmental prediction models, sensor, intelligent optimization algorithm

## Abstract

**Simple Summary:**

This study addresses the critical need for accurate prediction of key environmental factors—temperature, humidity, ammonia, and hydrogen sulfide—in pig houses, essential for pigs’ growth and health. Traditional methods face challenges in predictive accuracy and stability. We introduce an innovative OTDBO–TCN–GRU model, a hybrid framework combining the dung beetle algorithm, temporal convolutional network, and gated recurrent unit, enhanced by the Osprey optimization algorithm (OOA). This model synergistically merges DBO’s optimization power, TCN’s long-term dependency handling, and GRU’s proficiency in nonlinear sequence management, offering improved global detection capabilities. The OTDBO–TCN–GRU model showcases superior accuracy in environmental prediction, evident from its mean absolute error (MAE) of 0.0474, mean squared error (MSE) of 0.0039, and correlation coefficient of 0.9871. It significantly surpasses the traditional DBO–TCN–GRU and OOA models, reducing MAE and MSE by 37.2% and 66.7%, and 48.7% and 74.2%, respectively. Moreover, it outperforms mainstream models like GRU, LSTM, and XGBoost in terms of accuracy. This model significantly improves the forecasting of environmental conditions within pig houses, which is vital for maintaining optimal living conditions and ensuring the well-being of pigs.

**Abstract:**

Temperature and humidity, along with concentrations of ammonia and hydrogen sulfide, are critical environmental factors that significantly influence the growth and health of pigs within porcine habitats. The ability to accurately predict these environmental variables in pig houses is pivotal, as it provides crucial decision-making support for the precise and targeted regulation of the internal environmental conditions. This approach ensures an optimal living environment, essential for the well-being and healthy development of the pigs. The existing methodologies for forecasting environmental factors in pig houses are currently hampered by issues of low predictive accuracy and significant fluctuations in environmental conditions. To address these challenges in this study, a hybrid model incorporating the improved dung beetle algorithm (DBO), temporal convolutional networks (TCNs), and gated recurrent units (GRUs) is proposed for the prediction and optimization of environmental factors in pig barns. The model enhances the global search capability of DBO by introducing the Osprey Eagle optimization algorithm (OOA). The hybrid model uses the optimization capability of DBO to initially fit the time-series data of environmental factors, and subsequently combines the long-term dependence capture capability of TCNs and the non-linear sequence processing capability of GRUs to accurately predict the residuals of the DBO fit. In the prediction of ammonia concentration, the OTDBO–TCN–GRU model shows excellent performance with mean absolute error (MAE), mean square error (MSE), and coefficient of determination (*R*^2^) of 0.0474, 0.0039, and 0.9871, respectively. Compared with the DBO–TCN–GRU model, OTDBO–TCN–GRU achieves significant reductions of 37.2% and 66.7% in MAE and MSE, respectively, while the *R*^2^ value is improved by 2.5%. Compared with the OOA model, the OTDBO–TCN–GRU achieved 48.7% and 74.2% reductions in the MAE and MSE metrics, respectively, while the *R*^2^ value improved by 3.6%. In addition, the improved OTDBO–TCN–GRU model has a prediction error of less than 0.3 mg/m^3^ for environmental gases compared with other algorithms, and has less influence on sudden environmental changes, which shows the robustness and adaptability of the model for environmental prediction. Therefore, the OTDBO–TCN–GRU model, as proposed in this study, optimizes the predictive performance of environmental factor time series and offers substantial decision support for environmental control in pig houses.

## 1. Introduction

As modern animal husbandry progresses rapidly, the refinement of environmental management in pig houses has become essential in boosting productivity and upholding the well-being of animals [1,2]. Temperature and humidity, along with concentrations of ammonia and hydrogen sulfide, are the primary environmental factors influencing the growth and health of pigs in pig houses [3,4]. Accurate prediction of environmental factors is not only essential for animal growth and development [5] but also a cornerstone for intelligent and automated livestock management [6,7,8]. Effective environmental control can reduce resource wastage, optimize production costs, improve livestock health and productivity, and thus promote sustainable agriculture [9].

In order to improve the prediction accuracy of complex and dynamically changing environmental factors in piggery environments, researchers have carried out a variety of studies that range from traditional statistical methods to modern machine learning techniques [10,11,12]. The high degree of non-linearity, rapid dynamic changes, and strong data dependence of the swine barn environment have led to the fact that existing prediction methods often face problems of insufficient prediction accuracy and poor adaptability to environmental changes [13,14,15]. To address these challenges, researchers have explored various optimization algorithms and models [16,17,18].

For the pig house environment, Xie et al. [19] proposed an adaptive neuro-fuzzy inference system (ANFIS) to simulate human fuzzy thinking in order to deal with the high volatility, complexity, and non-linear relationships of the input parameters. This approach has shown to be a useful strategy in environmental control systems for pig houses. Meanwhile, in order to investigate the correlation between the indoor thermal environment and the gas concentration conditions and their characteristics, an innovative methodology incorporating triple correlation analysis and a multiple regression model for ammonia concentration was developed [20], which led to the identification of the CO_2_ concentration and outdoor temperature in the pig living space (PLS) as the determinants of the gas concentration and the thermal environment.

For algorithm optimization, Wang et al. [21] proposed a precipitation prediction model based on the coupling of empirical modal decomposition (EMD), dung beetle optimization (DBO) algorithm, and gated recurrent unit (GRU) neural network to improve the prediction accuracy. Zhu et al. [22] proposed a hybrid dung beetle search (QHDBO) algorithm to reduce the probability of falling into local optimum by improving the initial population distribution and introducing a convergence factor and dynamic equilibrium between the number of egg-laying and foraging dung beetles. In addition, Chen et al. [23] proposed a quasi-contrastive chaos-based mechanism WOA to overcome the original slow convergence speed of the whale optimization algorithm (WOA) in order to improve the algorithm’s convergence speed and balance the exploration and exploitation capabilities to help the algorithm jump out of the local optimum. D.S. in UAV 3D trajectory planning, an enhanced multistrategy dung beetle algorithm (EMSDBO) was used to optimize the trajectory quality [24].

For the mainstream prediction models, Yang et al. [25] proposed a hybrid model combining genetic algorithm (GA), variational modal decomposition (VMD), improved dung beetle optimization algorithm (IDBO), and bidirectional long- and short-term storage memory network based on attention mechanism (BiLSTM-A). Hui et al. [26] proposed a greenhouse environment prediction model based on the SSA-LSTM model, which significantly improved the accuracy of greenhouse environment prediction. Zhu et al. [27] explored an XGBoost-based method for greenhouse environment prediction and film-rolling decision making, which demonstrated the potential of machine learning in improving the efficiency of agricultural production. To solve these problems, researchers have explored various algorithms and models, such as support vector machines, neural networks, random forests, etc., but these methods still have limitations in dealing with time series data and capturing long-term dependencies [28,29,30,31,32,33].

Considering the above challenges, this study proposes a novel hybrid model, the improved dung beetle algorithm-temporal convolutional network-gated control recurrent unit (OTDBO–TCN–GRU)-based predictive optimization model for environmental factors in piggeries. The model aims to integrate the optimization finding ability of the dung beetle algorithm (DBO), the ability of temporal convolutional networks (TCNs) to capture long-term dependencies, and the high efficiency of gated control loop units (GRUs) to deal with non-linear sequences. In addition, the Osprey optimization algorithm (OOA) is introduced in this study to enhance the global search strategy of the dung beetle algorithm (DBO) [34], which further improves the accuracy and robustness of the prediction. This study delves into optimizing the integration of diverse algorithms to improve the accuracy of predicting environmental factors in pigsties, increasing the model’s responsiveness to environmental fluctuations, and precisely analyzing the time-series data of these factors. By implementing the OTDBO–TCN–GRU model, this study improves the prediction accuracy of key factors such as ammonia concentration to a certain extent and shows higher performance in comparison with existing models including DBO–TCN–GRU, OOA, GRU, LSTM, and XGBoost. Such improvements are crucial for accurately regulating the environment in pig houses.

## 2. Materials and Methods

### 2.1. Data Acquisition

**Figure 1:**
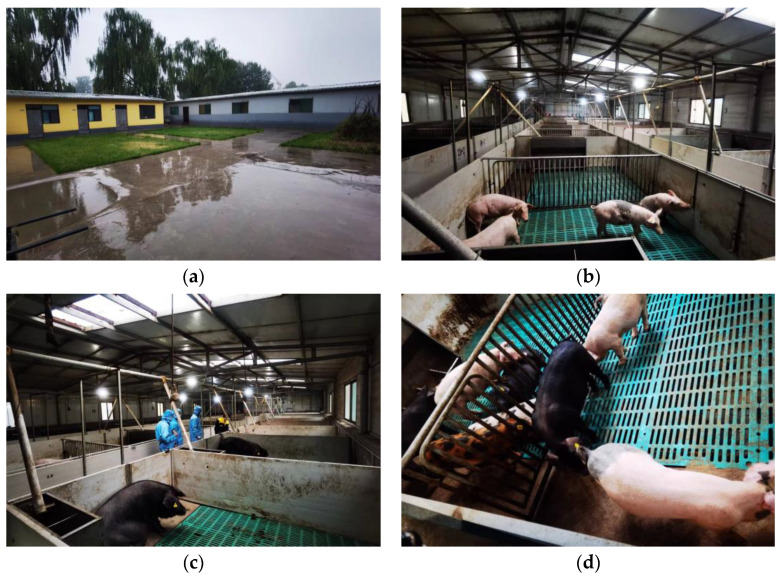
Real view of pig house environment. (**a**) Exterior view of pig house. (**b**) Internal view of the pig house. (**c**) Pig health checks. (**d**) Juvenile pigs.

**Figure 2:**
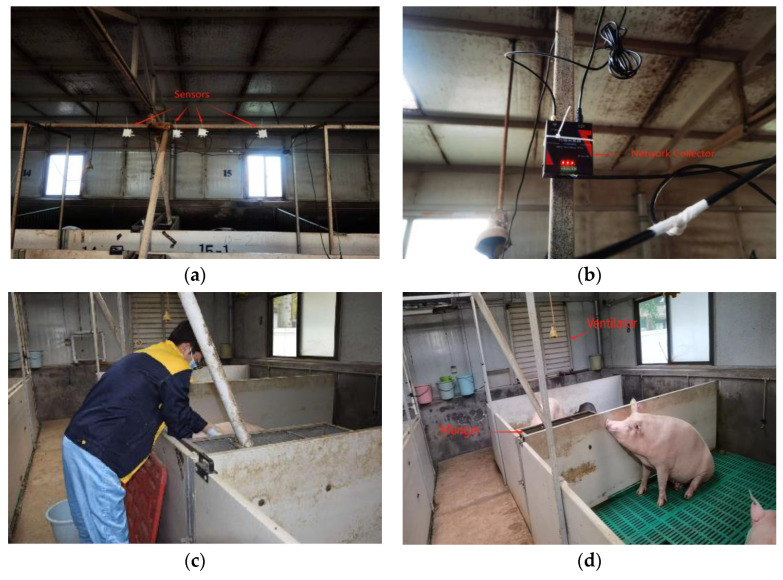
Sensor and ventilator position. (**a**) Environmental sensors. (**b**) Network collector. (**c**) Feeding pigs. (**d**) Manger and ventilator.

The pig industry is an important part of agriculture in many countries around the world, and concern for the health of pigs is also a sign of respect for animal welfare. To ensure the healthy growth of pigs, the impact of the environment on pigs is explored. In this study, we conducted a preliminary investigation in a swine facility at Shanxi Agricultural University, located in Taigu District, Shanxi Province, as shown in Figure 1. Image a is an exterior view of the pigsty, image b is an overview of the interior of the pigsty, image c is of researchers checking the health status of the pigs, and image d is of the pigs inside the house. The research was conducted using an environmental monitoring system manufactured by Shandong Renke Control Technology Co., Ltd., Jinan, China to collect environmental data in the house, which used a 4G network collector as the data recording and storage device, and RS485 communication to connect. Data metrics included indoor and outdoor temperature and humidity, levels of carbon dioxide, hydrogen sulfide, and ammonia, as well as outdoor wind speed. For a thorough and precise representation of the barn’s environmental conditions, sensors were strategically located in various undisturbed sections of the barn, enabling comprehensive data acquisition. The system’s overall configuration and sensor placement are illustrated in Figure 2.

The current structure of the pig house is designed with pouring column height cement as the external design, surrounded by aluminum alloy heat insulation and thermal insulation materials, with windows designed according to the partitioning of the columns, and the top of the house is made of translucent materials to ensure the daytime lighting. There are three fans at the end of the pig house for gas exchange inside and outside the house, and the ventilation of the fans was recorded during the test period.

The whole house is 26 m long and 12 m wide. The general parameters of the sensor are shown in Table 1. The sensor was installed in pens 5 and 15, respectively. Pen 5 monitored the environmental data of young pigs at a height of 1.5 m above the ground, and pen 15 monitored the environmental data of adult pigs at a height of 2.3 m above the ground. There were 115 pigs in the barn, of which 32 were adult pigs with an average weight of 110 kg, 55 were young pigs with an average weight of 45 kg, and the others were breeding sows and piglets. Because young pigs and adult pigs account for a large proportion of the entire growth cycle of pigs, this stage is preferred as the focus of the study. The outdoor sensors use the louver box and the wind speed sensors to collect the temperature, humidity, and wind speed of the pig outside the pig house at a height of 2.5 m. Outdoor sensors were selected from Shandong Renke Control Technology Co., Ltd., and the outdoor temperature, humidity, and wind speed were collected at a height of 2.5 m outside the pig house using a louver box and a wind speed sensor. All sensors collected data every 30 min from 1 July 2023 to 15 August 2023, and a total of 33,865 pieces of data were collected.

### 2.2. Data Processing

#### 2.2.1. Data Outlier Handing

Subject to sudden changes in the environment and occasional anomalies in the sensor when saving data [35], and to ensure the continuity of time, it is necessary to modify the anomalous data to reduce the impact on the experimental results. This experiment uses the 3σ principle to deal with noisy data [36]. The absolute value of the residual error in the experimental data vi > 3σ is judged as abnormal based on the normal distribution function as shown in Equation (1).

(1) $$f(x)=\frac{1}{\sqrt{2\pi }}e^{\left(-\frac{x^{2}}{2}\right)}$$

#### 2.2.2. Data Normalization

In order to eliminate the influence of the magnitude of different dimensional data on the prediction model, the study adopts min–max deviation normalization [37,38], which linearly transforms the original data so that the results are mapped between [0.0 and 1.0] to improve the model convergence speed and prediction accuracy, as shown in Equation (2).

(2) $$X_{\mathrm{norm}}=\frac{X_{i}-X_{\min}}{X_{\max}-X_{\min}}$$

In Equation (2), *X_i_* represents the *i*th measurement, *X*_max_ represents the maximum value of the measurement, and *X*_norm_ represents the normalized measurement.

#### 2.2.3. Data Noise Reduction

The Stavisky−Golay filter is a smoothing technique based on polynomial fitting [39]. Unprocessed data, when used, will affect the results of the experiment. The filter involves fitting a local polynomial to the signal and then replacing the original signal with the fitted polynomial for smoothing.

In this study, we used Python [40] (Edition 2021.3) as the main programming language and PyCharm development tool to construct and test the OTDBO−TCN−GRU hybrid model, in which the algorithm was completed to run efficiently using deep learning frameworks such as Pandas, Scikit-learn, and TensorFlow. In addition, to ensure the quality of the data and the accuracy of the experiments, we used SPSS [41] for data processing and analyses. The graphing and visualization tools of SPSS support exploratory data analysis, which is remarkable for discovering patterns, trends, and outlier variations in the data. This was useful in determining the type of predictive model that would be appropriate for the data.

### 2.3. Predictive Modelling of Piggery Environments

#### 2.3.1. Dung Beetle Optimization (DBO) Algorithm

Dung beetle optimizer (DBO) is a new population intelligence optimization algorithm proposed in 2022 [42]. The algorithm is inspired by the behaviors of dung beetles: foraging, ball rolling, dancing, breeding, and stealing behaviors, and the algorithm is characterized by high convergence speed and high accuracy. The dung beetle optimization algorithm achieves parameter optimization by performing corresponding operations with dung beetles of different behavioral types. It is specifically divided into four strategies.

Strategy 1: Ball rolling

Dung beetles usually use the sun as a guide to ensure that they roll the dung ball on a straight path, but natural factors such as light intensity and wind can affect dung beetle ball rolling while traveling. The position of the dung beetle when rolling the ball is updated based on Equations (3) and (4).

(3) $$x_{i}^{t+1}=x_{i}^{t}+\alpha \times k\times x_{i}^{t-1}+b\times \Delta x$$

(4) $$\Delta x=|x_{i}^{t}-X^{w}|$$

In the above formula, *t* represents the current number of iterations, $x_{i}^{t}$ denotes the first iteration of t positional information of the *i*th dung beetle at the time of the second iteration, *α* is a natural coefficient indicating whether or not it has deviated from its original direction, assigned as −1 or 1 according to the probabilistic method, k∈(0.0,0.2) denotes the deflection coefficient, b∈(0.0,1.0) denotes a fixed parameter, and $X^{w}$ denotes the global worst position. Δx is used to simulate changes in light intensity.

When a dung beetle encounters an obstacle that obstructs its rolling path, it needs to reposition itself by dancing to find a new rolling route. To simulate this dancing behavior, a tangent function can be used to obtain the new rolling direction. After determining the new rolling direction, the dung beetle will continue to roll the dung ball. The new position is updated according to Equation (5).

(5) $$x_{i}^{t+1}=x_{i}^{t}+\tan\theta |x_{i}^{t}-x_{i}^{t-1}|$$

In the above formula, the θ∈[0,π] denotes the angle of deflection, when θ is equal to 0, $\frac{\pi }{2}$, or π. The position of the dung beetle is not updated.

Strategy 2: Dung beetle with ovoid ball (rood ball)

In nature, dung beetles roll dung balls to a safe place to hide them for female dung beetles to lay their eggs in a suitable environment. The spawning area of the female dung beetle is modeled in the algorithm, resulting in the calculation of Equations (6) and (7).

(6) $$Lb^{*}=\max(X^{*}\times (1-R),Lb)$$

(7) $$Ub^{*}=\min(X^{*}\times (1+R),Ub)$$

where $X^{*}$ is the current local optimum position, and $Lb^{*}$ and *U*$b^{*}$ are the lower and upper bounds of the spawning area, respectively. Inertial weights $R=1-t/T_{max}$, $T_{max}$ denote the maximum number of iterations, and Lb and Ub represent the upper and lower bounds of the optimization problem, respectively. the change of spawn balls during the iteration process is defined as Equation (8).

(8) $$B_{i}^{t+1}=X^{*}+b_{1}\times (B_{i}^{t}-Lb^{*})+b_{2}\times (B_{i}^{t}-Ub^{*})$$

where $B_{i}^{t}$ is the *t*th iteration of i, which is the position information of an ovoid, and $b_{1}$ and $b_{2}$ are uncorrelated random vectors representing the number of dimensions of the optimization problem.

Strategy 3: Young dung beetles foraging (small dung beetle)

The eggs laid by female dung beetles gradually grow into baby dung beetles, and some of the mature baby dung beetles go in search of food, with the optimal foraging area defined by Equations (9) and (10).

(9) $$Lb^{b}=\max(X^{b}\times (1-R),Lb)$$

(10) $$Ub^{b}=\min(X^{b}\times (1+R),Ub)$$

where $Lb^{b}$ and $Ub^{b}$ denote the lower and upper boundaries of the optimal foraging area, respectively, and b is the global optimal position. The position update of the young dung beetle is derived from Equation (11).

(11) $$x_{i}^{t+1}=x_{i}^{t}+C_{1}\times (x_{i}^{t}-Lb^{b})+C_{2}\times (x_{i}^{t}-Ub^{b})$$

In the above formula, $x_{i}^{t}$ is the *t*th iteration of i, which is the location information of the young dung beetle; $C_{1}$ is a random number that follows a normal distribution; $C_{2}\in (0.0,1.0)$, is the random vector.

Strategy 4: Stealing behavior (THIEF)

Some dung beetles known as thieving dung beetles will steal other dung beetle egg balls and food. The location of the thieving dung beetle is updated by Equation (12).

(12) $$x_{i}^{t+1}=X^{b}+S\times g\times (|x_{i}^{t}=X^{*}|+|x_{i}^{t}-X^{b}|)$$

In the above equation, $X^{b}$ is the optimal food source, g is a random vector of size 1×D a random vector obeying a normal distribution, and *S* denotes a constant value.

**Figure 3:**
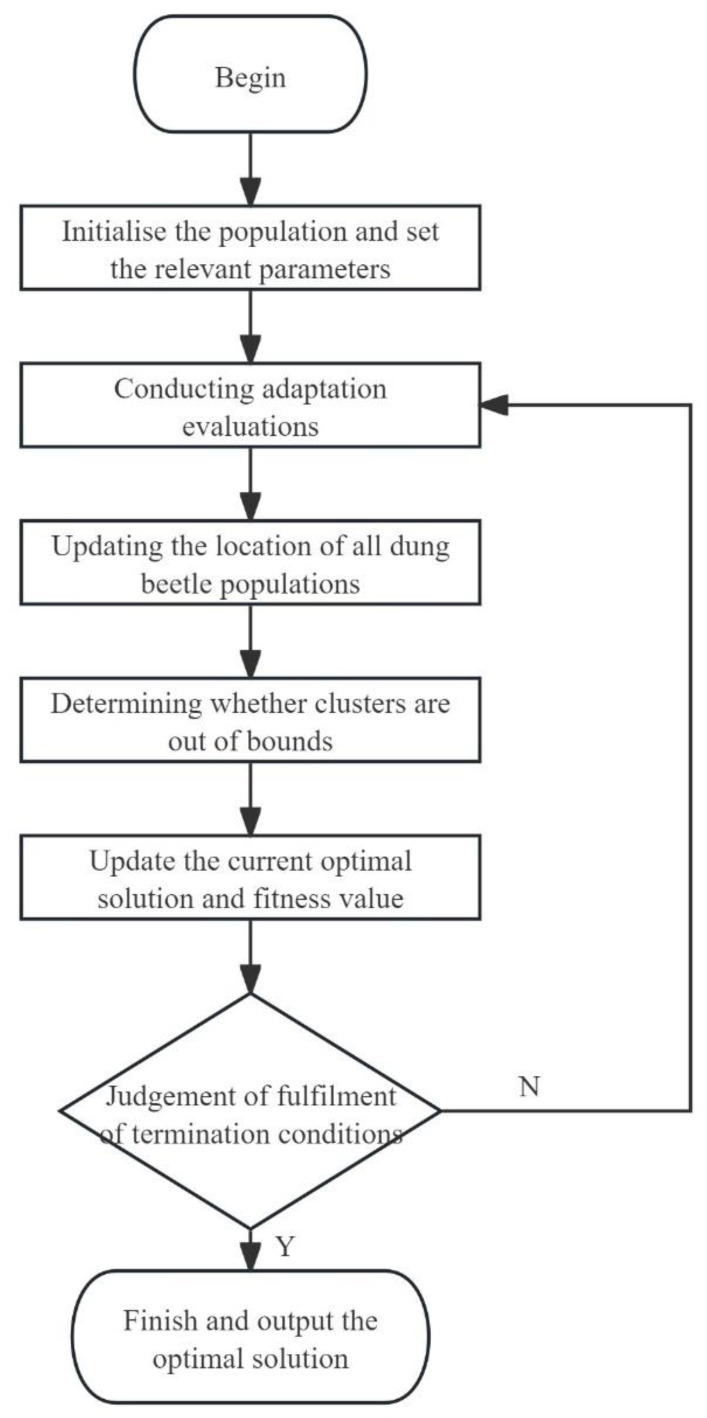
Logic flowchart of DBO algorithm.

Based on the dung beetle optimization algorithm strategy, a flowchart is constructed for the main logic of the algorithm, as shown in Figure 3.

#### 2.3.2. TCN−GRU

Temporal convolutional networks (TCNs) incorporate two different neural network architectures of gate control recurrent units (GRUs), TCNs are a type of convolutional neural networks for processing time series data [43]. Causal convolution with dilation convolution as a key feature ensures that only information from the current moment and its predecessors is applied when predicting the output at the current moment, while dilation convolution is able to cover longer input sequences by increasing the spacing between convolution kernels to capture longer-term dependencies. GRU, a variant of recurrent neural networks [44], has an excellent performance in capturing short-term dependencies in time series. This combination combines the long-term learning capability of TCN with the temporal dynamic modeling capability of GRU. It is suitable for the present experimental situation.

In TCN−GRU structure, TCN firstly processes the input sequence to capture the long-term dependencies and secondly GRU layer receives the output of TCN to further process the short-term dynamics in the time series TCN is used to represent the dilated convolution as in Equation (13).

(13) $$F(t)=(X{*}_{d}W)(t)$$

where ${*}_{d}$ denotes the dilated convolution operation, X is the input sequence, W is the convolution kernel, and t is the time step.

The update gate and reset gate of GRU can be expressed by the following equation:

Update the door:

(14) $$z_{t}=\sigma (W_{z}*x_{t}+U_{z}*h_{t-1})$$

Reset the door:

(15) $$r_{t}=\sigma (W_{r}*x_{t}+U_{r}*h_{t-1})$$

New status:

(16) $$\tilde{h}=\tanh(W*x_{t}+U*(r_{t}⊙h_{t-1}))$$

Output status:

(17) $$h_{t}=(1-z_{t})⊙h_{t-1}+z_{t}⊙{\tilde{h}}_{t}$$

In its formula, the σ is the sigmoid activation function, ⊙ denotes element-by-element multiplication, *W* and *U* are weight matrices, and $h_{t}$ is the hidden state at the current moment.

GRU is good at dealing with short-term dependence and dynamic changes in time series data, and is very effective in predicting short-term transformations as well as capturing unexpected events, while the TCN−GRU combination can learn features better. Compared with traditional recurrent neural networks (RNNs), the fusion model has better gradient retention propagation and reduces the problem of gradient vanishing in gradient explosion.

### 2.4. Incorporating Multiple Strategies to Improve DBO

The four types of DBO algorithms have the characteristics of strong Osprey optimization algorithm ability and fast convergence speed, but at the same time, there is also the problem of imbalance between global exploration and local exploitation ability. For the problem of poor global exploration of DBO, the Osprey optimization algorithm strategy is used by using the fusion of the Osprey optimization algorithm, while for the problem of imbalance in the local exploitation ability, the improvement of the sample sampling strategy and the distribution of the perturbation to avoid DBO algorithms to fall into the local optimal solution are employed. In the present study, three improvement points are proposed to improve the model accuracy for the shortcomings of the model.

#### 2.4.1. Fusion Osprey Optimization Algorithm (OTDBO)

The global exploration strategy of the Fish Eagle Osprey optimization algorithm (OOA) in the first stage is used to replace the position update formula of the original dung beetle algorithm in the ball−rolling stage [45]. The global exploration strategy of the Fish Eagle optimization algorithm can make up for the drawbacks of the dung beetle algorithm which only relies on the worst value in the rolling behavior, cannot communicate with other dung beetles in time, and has more parameters. The global exploration strategy of the Fish Eagle algorithm is used to randomly detect the position of one of the dung balls and roll it, which improves the global detecting ability.

#### 2.4.2. Latin Hypercube Initialization Population

Latin hypercube sampling is a multidimensional stratified sampling technique proposed by McKay et al. It can efficiently sample between the distribution of variables, and its essence is to divide the interval [0, 1] into N equally spaced non-overlapping sub-intervals, and carry out independent equal probability sampling on each sub-interval to ensure that the sampling points are uniformly distributed throughout the entire distribution. Random sampling obeys a uniform distribution in the interval [0, 1], and in the case of a small number of samples, the random distribution does not spread the samples well over the entire interval. Unlike random sampling, Latin hypercube sampling ensures that the variables are covered over the entire distribution space.

#### 2.4.3. Adaptive t-Distribution Perturbation Strategy

The purpose of the adaptive t-distribution perturbation strategy is to increase the algorithm’s versatility and exploration capabilities during the search process [46]. The adaptive t-distribution perturbation strategy is used when t(n→∞)→N(0,1), where N(0,1) is a Gaussian distribution, and the Gaussian variant is characterized by a high capacity for local exploitation; and when t(n=1)=C(0,1), where C(0,1) is a Cauchy distribution, and the Cauchy variant is characterized by a strong global search capability [47]. The adaptive t-distribution fuses the Gaussian and Cauchy distributions, which gives the algorithm a good global exploitation ability in the early iteration, a good local exploration ability in the late iteration, and improves the convergence speed of the algorithm. The specific position is updated as in Equation (18).

(18) $$X_{i+1}^{j}=X_{b}^{j}+t(C\_iter)\times X_{b}^{j}$$

In the above equation, $X_{i+1}^{j}$ is the optimal solution after adaptive t-distribution variational perturbation in the first j dimension, $X_{b}^{j}$ is the position of the optimal solution before the variational perturbation in dimension j dimension, iter is the number of iterations, and the number of iterations is used as the parameter of the degrees of freedom of the t-distribution.

**Figure 4:**
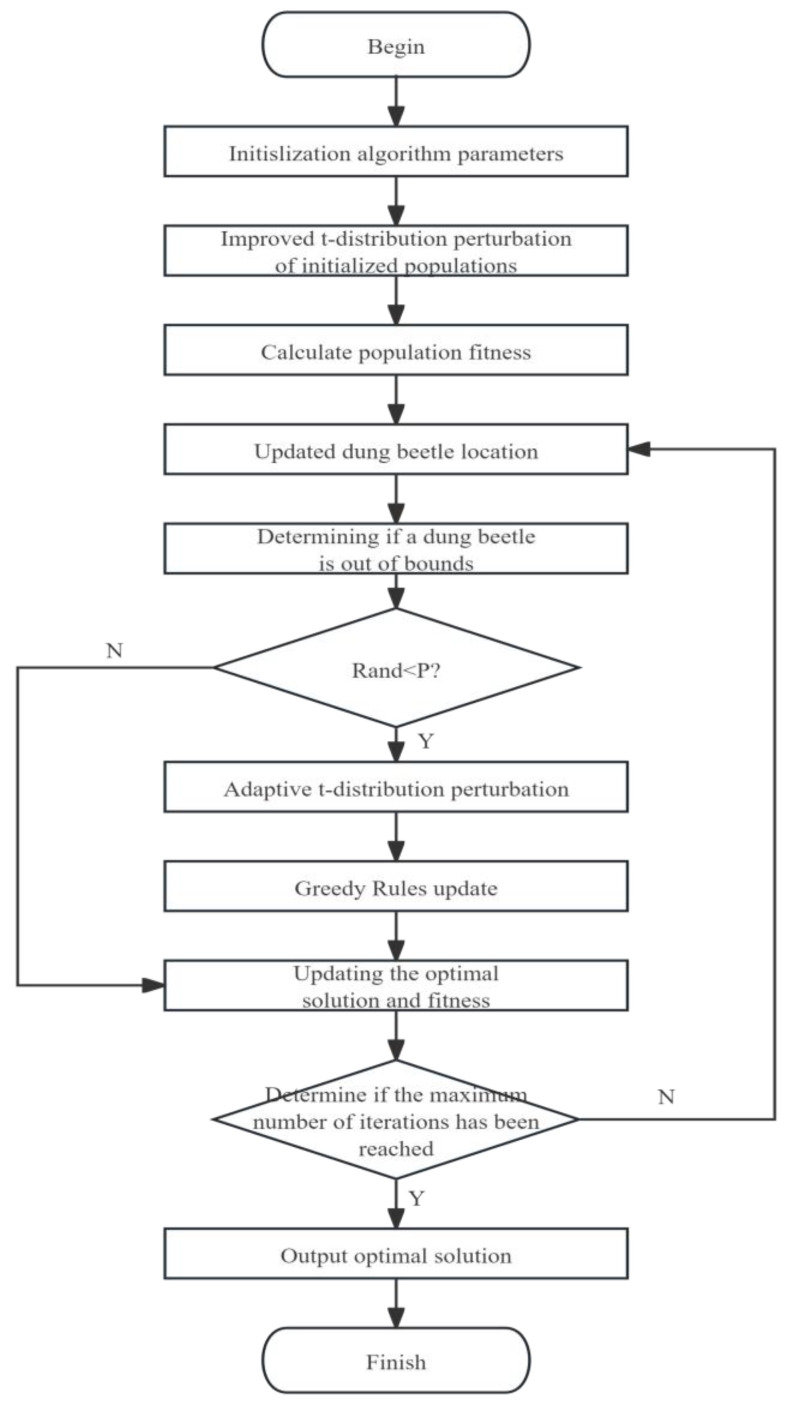
Flowchart of the DBO with the introduction of t-perturbation.

At the beginning of the iteration, the t-distribution perturbation resembles the Cauchy variation, and when the algorithm has good global exploration ability, it increases the diversity of the population, and the ability to jump out of the local optimum is enhanced. As the number of iterations increases, the t-distribution perturbation approximates the Gaussian variation, which improves the algorithm’s ability of local exploitation, and its perturbation strength for the whole population transforms from strong to weak. By introducing adaptive t-distribution mutation as an improved search strategy, the optimization performance of the algorithm can be effectively enhanced, which is conducive to improving the ability of the algorithm to escape from the local optimum. The greedy rule is also added to determine whether to update the position by comparing the fitness values of the old and new positions. The flow chart of the dung beetle optimization algorithm with the addition of t-distribution mutation is shown in Figure 4.

### 2.5. Evaluation Indicators

In this study, the coefficient of determination (*R*^2^), the root means square error (*MSE*), and the mean absolute error (*MAE*) are used as a measure of the model’s accuracy and predictive power. The larger the *R*^2^, the smaller the *MSE* and *MAE*, indicating the more accurate the prediction model. In addition, m is the number of samples, $y_{i}$ is the true value, and y^i is the predicted value. The evaluation indexes are shown in Equations (19)–(21).

(19) R2=1−∑i (y^i−yi)2∑i (yi¯−yi)2

(20) MSE=1m∑i=1m(yi−y^i)2

(21) MAE=1m∑i=1m|(yi−y^i)|

## 3. Results

### 3.1. Pearson Correlation Analysis

**Figure 5:**
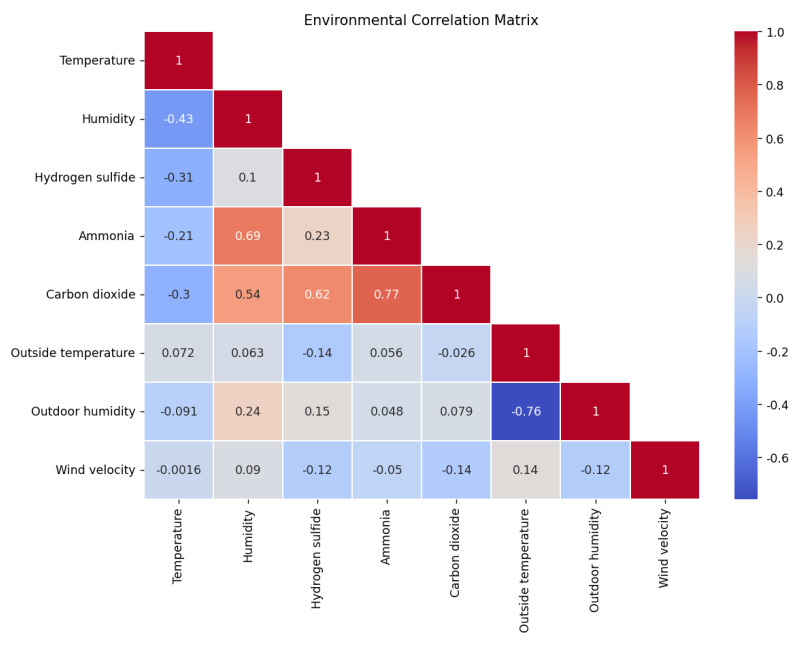
Internal environmental correlation matrix.

Pearson’s correlation coefficient is commonly used to accurately measure the closeness of the relationship between two variables. [−1, 1] Pearson explains the influence of features on the prediction model through the attribution value of the features; a positive value indicates that the features of the two variables reflect a positive effect on the model prediction, and vice versa, a negative value has a negative effect on the model prediction, and the larger the value is, the more obvious the influence effect is. As shown in Figure 5.

As can be seen from Figure 5, different colors distinguish the degree of correlation: the deeper the degree of red represents a stronger positive correlation, and the deeper the degree of blue represents a stronger negative correlation. Taking ammonia concentration as an example, the Pearson coefficient calculates the degree of correlation between the change of ammonia concentration factor and temperature, humidity, and other factors in the pig house, and then determines which data have the greatest influence on ammonia concentration among the non−chronological data affecting ammonia concentration, so as to select the appropriate data for input and prediction. From the table, it can be seen that the environmental factor that affects the ammonia concentration the most is the carbon dioxide concentration, followed by indoor humidity as well as outdoor humidity, while the hydrogen sulfide concentration and outdoor temperature correlate with the ammonia concentration in general, and the indoor temperature shows a negative correlation with the wind speed.

### 3.2. OTDBO−TCN−GRU Model Optimization and Training

In order to build the prediction model of the pig house environment more accurately, 33,865 data collected during the test period were processed and divided into a training set and test set in the ratio of 8:2. A 12-parameter input and a single-parameter output prediction model was constructed to predict each parameter in turn.

The optimized training parameters were as follows: the number of dung beetle populations in OTDBO was 3, the learning rate was set to 0.001, the number of training iterations was 50, the lower boundary hidden layer neuron and its filter search range were [1, 16], the upper boundary hidden layer neuron and its filter search range was [32, 128], and the number of training rounds for the model was 200. The training records were recorded for the adjusted two-layer filters and neurons in each iteration number of neurons to derive the final fitness. The training was stopped when the fitness did not change for three consecutive rounds, and the optimization results are shown in Table 2. After seven training rounds, the model is almost close to a steady state, and optimal fitness is reached during eight rounds of training.

**Figure 6:**
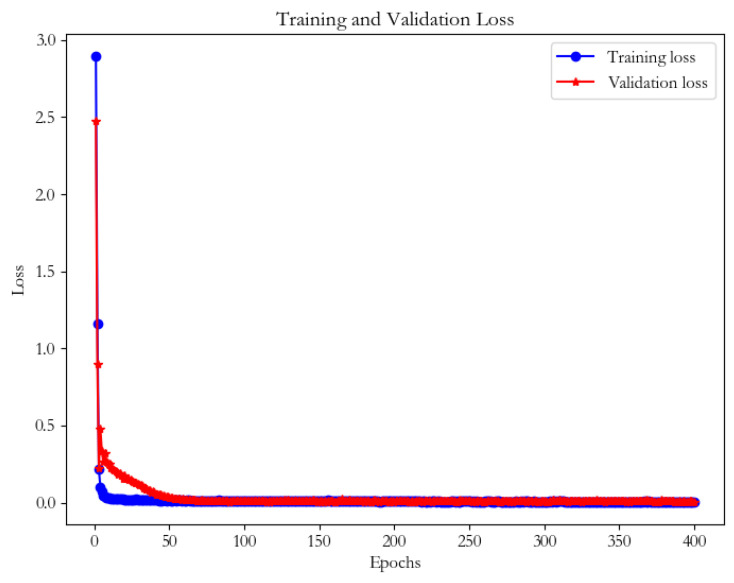
Training validation loss graph.

In the OTDBO−TCN−GRU model training, it can be seen by the training loss and validation loss curves in Figure 6 that both losses significantly decrease and stabilize with the increase of the training cycles (Epochs). The training loss decreases rapidly in the first few cycles, which implies that the model learns quickly from the data. As the number of cycles increases, the training loss stabilizes in almost 10 rounds of training and is almost close to 0. Meanwhile, the validation loss drops to a low level and remains stable in almost 60 rounds of training. This indicates that the model gradually improves during the learning process without overfitting, and therefore the model has strong generalization as well as prediction ability to the data.

### 3.3. Comparison of Real and Predicted Values

**Figure 7:**
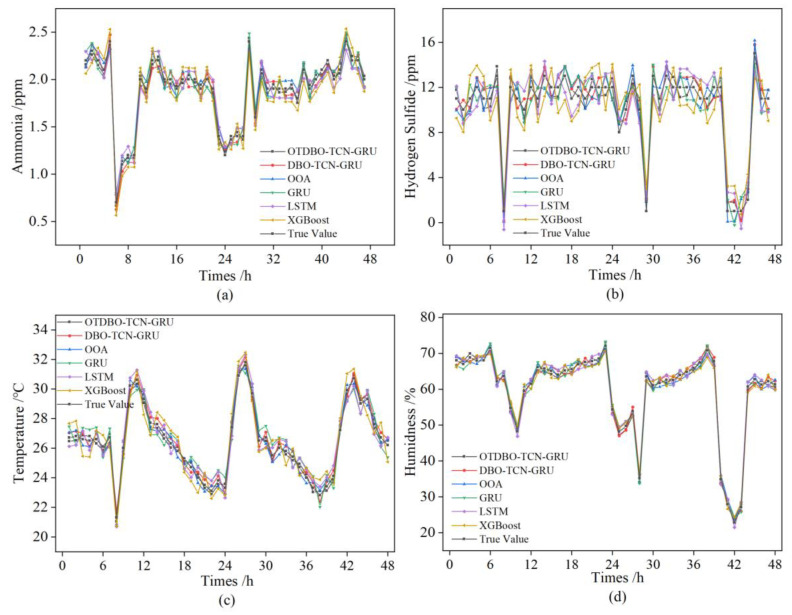
Model prediction comparison chart. (**a**) Comparison of ammonia prediction models. (**b**) Comparison of Hydrogen Sulphide Prediction Models. (**c**) Comparison of temperature prediction models. (**d**) Comparison of humidity prediction models.

Figure 7 shows the comparison of the prediction result errors of the mainstream environmental prediction models such as OTDBO−TCN−GRU, DBO−TCN−GRU, OOA, GRU, LSTM, and XGBoost. The horizontal axis of the figure represents 48 h of continuous monitoring of selected piggeries, and the vertical axis represents the amount of environmental data states. From the figure, it can be seen that XGBoost and LSTM models predicted poorer results, DBO−TCN−GRU, OOA, and GRU have a higher overall fit, but in the prediction of hydrogen sulfide encountered in the case of environmental mutations, the accuracy of the prediction declined, and the prediction error mostly exceeded ±0.3 mg/m^3^, concentrating in the range of 0.3–0.5 mg/m^3^; the prediction difference was larger, and the result was less satisfactory. The improved OTDBO−TCN−GRU is not affected by environmental mutations. It can be concluded that the prediction model based on OTDBO−TCN−GRU has higher accuracy and the prediction results are closer to the actual data of the pig house environment.

### 3.4. Comparison of Model Performance

According to the results in Table 3, taking the prediction of ammonia concentration as an example, we can conclude that the OTDBO−TCN−GRU model performs optimally in terms of prediction accuracy, with an MAE of 0.0474, an MSE of 0.0039, and an *R*^2^ of 0.9871. This means that the model has a small average absolute error between the predicted value and the actual value, the mean square error is also very small, and the *R*^2^ is close to 1, which indicates that the model can explain the changes in the actual data more accurately.

Compared with the DBO−TCN−GRU model, the OTDBO−TCN−GRU model achieved improvements in all metrics, with a 43.2 percent reduction in MAE and a 66.7 percent reduction in MSE, along with a 2.5 percent improvement in *R*^2^. This indicates that the OTDBO−TCN−GRU model has better predictive accuracy and stability, and can more accurately capture trends and patterns of change in the data.

In addition, the OTDBO−TCN−GRU model also showed significant advantages compared with the GRU and LSTM models. Compared with the GRU and LSTM models, the OTDBO−TCN−GRU model reduces the MAE by 48.7% and 51.6%, the MSE by 23.3% and 81.2%, and the *R*^2^ improves by 3.6% and 5.5%, respectively. The OTDBO−TCN−GRU model is capable of predicting the target variables more accurately and has a better performance in terms of reducing prediction error compared with the GRU and LSTM models with better performance.

The OTDBO−TCN−GRU model is even a significant improvement compared to the OOA and XGBoost neural network models. The OTDBO−TCN−GRU model reduces the MAE by 48.9% and 64.4%, the MSE by 76.1% and 89.6%, and the *R*^2^ improves by 4.6% and 10.9%, respectively, compared to the OOA neural network model. The OTDBO−TCN−GRU model has advantages in capturing the nonlinear relationships and complex patterns of the data, and is able to predict the target variables better compared to OOA and XGBoost neural network models.

**Figure 8:**
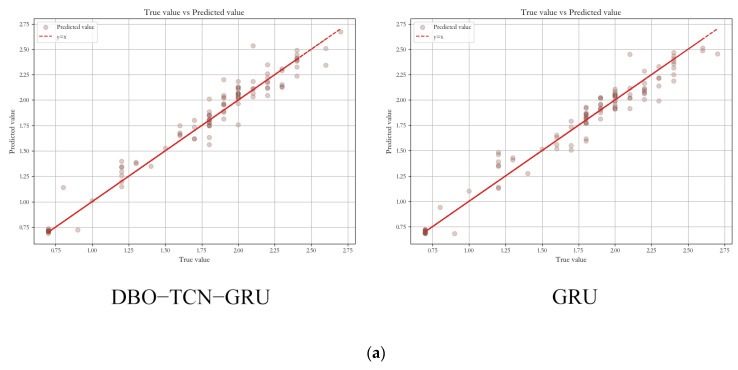

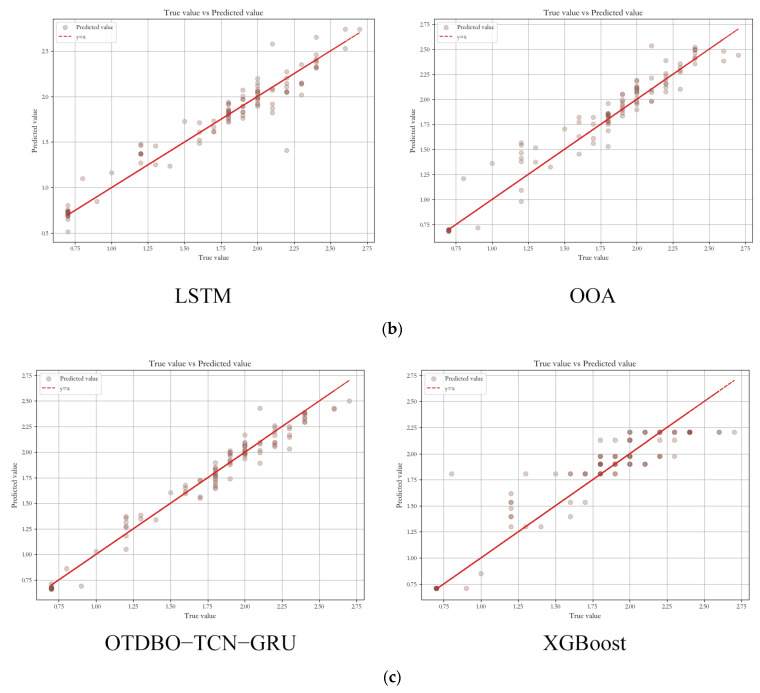
Model comparison scatter plots. (**a**) Comparison of DBO−TCN−GRU and GRU model prediction scatter plots. (**b**) Comparison of LSTM and OOA model prediction scatter plots. (**c**) Comparison of OTDBO−TCN−GRU and XGBoost model prediction scatter plots.

As shown in the scatter plot in Figure 8, the x-axis represents the true values and the y-axis represents the predicted values. From the scatter distribution in the graph, most of the points are located at or near the y = x line, which indicates that for most of the data points, the predicted values of these models are quite close to the true values. This trend indicates that the models are predicting with a high degree of accuracy. From the point comparison, it can be seen that the OTDBO−TCN−GRU model points are most concentrated on the y = x line, followed by the DBO−TCN−GRU model. In contrast, XGBoost and LSTM point locations are the most dispersed, i.e., the prediction results are poorer, and the model’s weak ability to cope with changes in the environment has limitations. More so, the OTDBO−TCN−GRU model has a strong coping ability for global prediction as well as environmental changes, and the prediction results are more accurate.

### 3.5. Influence of Outdoor Temperature on Predictions

A controlled experiment was carried out to address the effect of outdoor temperature on the experiment. The model was trained with the presence or absence of outdoor temperature as a variable for ammonia prediction. The results of the test are shown in Table 4. From the information in the table, it can be seen that removing the outdoor temperature changes the results predicted by the model, with *R*^2^ improving by 0.1%, MAE decreasing by 2.3%, and MSE decreasing by 7.6%. It is concluded that the prediction accuracy will be improved but the enhancement is not significant.

## 4. Discussion

### 4.1. OTDBO−TCN−GRU Compared to Other Models

In this study, we propose a dung beetle optimization algorithm based on improved dung beetle optimization algorithms to complete the study of predicting the environment of indoor pig barns. This study compares the results of six algorithms: OTDBO−TCN−GRU, DBO−TCN−GRU, OOA, LSTM, GRU, and XGBoost. Firstly, all six algorithms are considered to be the current dominant environmental prediction models, and all of them have an excellent performance in time-series prediction, which is crucial for scenarios in which environmental changes are monitored continuously. Second, they represent design philosophies and performance characteristics that perform differently in prediction in this domain. For example, DBO, inspired by the behavior of dung beetles in nature, is suitable for solving optimization problems, especially when the search space is complex or the problem is poorly defined; including resource allocation, scheduling, etc., it is suitable for global search scenarios. OOA simulates the hunting behavior of fish eagles, and the optimized search strategy is also advantageous for global optimization. LSTM and its variant GRU are able to capture long−term temporal dependence, which is an improvement on the gradient vanishing problem. XGBoost is an efficient end−to−end boosted tree algorithm, which is able to automatically deal with the missing data, and has a strong generalization ability.Finally, TCN provides an efficient way to capture the time dependence of sequence data with good parallel computing capability.

Conclusions were drawn for the prediction of multiple environmental factors in the house separately. Taking ammonia as an example, compared with the GRU and LSTM models, the OTDBO−TCN−GRU model reduced the MAE by 48.7% and 51.6%, reduced the MSE by 23.3% and 81.2%, and improved the *R*^2^ by 3.6% and 5.5%, respectively. Compared to its predecessors using EMD−LSTM prediction [48] and CNN−GRU prediction of environments [49], it has some improvement in prediction accuracy for multifactor and complex time series data.

OTDBO−TCN−GRU as a hybrid algorithm can be studied and compared with its predecessors to gain insight into the performance improvement of the new algorithm. The advantages of the OTDBO−TCN−GRU model can be found in more depth. Firstly, for capturing the time dependency in the data, the TCN structure can capture the long-term dependency well, which is important for predicting the trend and periodicity of environmental changes. Secondly, the fusion of the GRU structure can better predict the nonlinear data and improve the prediction accuracy. DBO algorithm has limitations in dealing with highly dynamic data and global optimization, while OTDBO fused with the OOA algorithm can greatly improve the global search ability and prediction accuracy. This fusion approach can better combine the data characteristics from data processing to the training process and can have better results.

### 4.2. Deficiencies of the Model and Future Plans

However, as a hybrid model despite having better prediction results in forecasting, it also has certain disadvantages and drawbacks. The increased number of parameters in the hybrid model makes tuning and training more difficult. At the same time, the large model size also means that the computational cost may increase significantly as it requires running and coordinating multiple algorithms at the same time. This may require more computational resources such as CPU time and memory.

In view of the current problems of the above models, it is the focus of future work to ensure that the prediction results are improved while simplifying the structure of the models. It is expected that the hybrid model can automatically adjust its structure and parameters according to the characteristics of the data, thus reducing the need for manual parameter adjustment and achieving a kind of adaptive ability. We should also include information on pig weight in future research. Pigs have different environments and ideal conditions during their growth stages, and these conditions have a great relationship with their body weight. At present, we have conducted prediction studies on young (9 to 16 weeks) and adult (17 to 24 weeks) pigs, which is a limitation and also needs to be improved in subsequent experiments. In future studies, we will carry out experiments on pigs of all ages, while monitoring the body weight information of pigs and modeling the body weight factor with the growth status to reach more comprehensive conclusions.

## 5. Conclusions

In this study, we propose an improved dung beetle optimization algorithm incorporating OOA and an OTDBO−TCN−GRU prediction model for pig barn environments. Experiments were carried out by using the barn carbon dioxide, temperature and humidity, ammonia, and hydrogen supplied, and environmental data were obtained from sensors as the datasets, which were processed by data outliers and noise reduction. Comparative evaluation of the effectiveness of environmental prediction using various models and methods led to the following conclusions:

- The Pearson correlation analysis experiments showed that all indoor gases are negatively correlated with temperature. Ammonia is most affected by humidity, and there is a small effect of outdoor temperature on ammonia prediction accuracy, and the removal of outdoor temperature improves prediction accuracy.
- The training loss in the OTDBO−TCN−GRU model training tends to stabilize at nearly 10 rounds of sub-clocks, almost close to 0, and the validation loss tends to stabilize at nearly 60 rounds of sub-clocks, which indicates that the model has a strong ability to generalize the data as well as stability.
- The improved OTDBO−TCN−GRU model compares with other algorithms to predict environmental gases with an error of less than 0.3 mg/m^3^, and has less impact on sudden environmental changes, which indicates that the model is robust and adaptable to environmental prediction.
- Compared with the traditional neural networks such as GRU, OOA, and LSTM, the MAE was reduced by 48.7%, 49.0%, and 51.6%, the MSE was reduced by 74.2%, 76.1%, and 81.2%, and the *R*^2^ was improved by 3.7%, 4.6%, and 5.5%, respectively, in the prediction. The model’s error with respect to the measured values was also investigated, and the OTDBO−TCN−GRU model achieved significant performance enhancement in predicting ammonia concentration, hydrogen sulfide concentration, and temperature and humidity compared to the GRU and LSTM models, reducing the percentage of MAE and MSE. It indicates that the OTDBO−TCN−GRU model performs more accurately and reliably in these prediction tasks.

In summary, the OTDBO−TCN−GRU model showed high accuracy and low peak error in the prediction of the pig house environment, and the model provides a theoretical basis for the application of precise regulation and damage warning for the indoor environment of pig houses, and provides useful decision support for the regulation of pig house environment.

Pig barn environment prediction technology has shown great potential for a variety of applications in the livestock industry, including feed conversion, disease detection, intelligent feeding, and precision breeding. Accurate prediction of pig housing environment helps to improve growth rate and feed conversion efficiency, reduce mortality and disease incidence, and ultimately improve overall productivity; secondly, reducing unfavorable environments and stressful behaviors plays an important role in ensuring the welfare of pigs, and is an aspect that is now receiving increasing attention in the livestock industry; practical breeding combined with environmental prediction technology supports sustainable development of livestock farming by reducing energy consumption and mitigating the impact on the environment (reduction of greenhouse gas emissions), supporting the sustainable development of animal husbandry; in terms of intelligent farming, with the development of IoT, big data, and artificial intelligence technologies, pig house environment prediction can become part of an intelligent farming system, which automatically adjusts the farming environment through real-time data collection, analysis and prediction to achieve highly automated and intelligent farming management. In summary, pig house environment prediction has far-reaching significance for realizing healthy pig farming, which not only concerns the health and welfare of pigs but also affects farming efficiency, economic benefits, and environmental sustainability. By using scientific methods and technologies to predict and manage the pig house environment, the farming industry can achieve more efficient, sustainable, and humane farming practices.

In future studies, we will further refine and expand the determination of the relationship between body weight measurement and environmental factors in pigs. Body weight is one of the most important indicators of the health and growth of pigs, which has both indirect and direct effects on pigs. Changes in the environment affect the changes in body weight, which in turn affects the nutritional status, growth rate, and possible health problems of pigs. In addition, considering the possible differences in the environmental sensitivity of pigs at different growth stages, we plan to produce a new dataset by subdividing the data by age groups in order to more accurately assess the effects of environmental changes on the body weight and health of pigs at different developmental stages. The next research task is to lighten the model treatment, to achieve the advantages of maintaining the current model while eliminating some unnecessary components to reduce the time and cost of training.

We aim to reveal the complex relationship between pig weight gain and environmental factors, so as to provide a scientific basis for the precise regulation of the pig house environment and optimize pig breeding management strategies. Future studies will also explore the potential mechanisms of intervention in pig weight gain through environmental regulation and the long-term effects of these interventions on the overall health and welfare of pigs, thus providing more scientific and humane guidance for animal husbandry practices. This will not only improve farming efficiency and performance, but also promote animal welfare and contribute to the sustainable development of the livestock sector.

## Figures and Tables

**Table 1:** Parameters of environmental sensors in pig house.

Parametric	Model	Range	Resolution	Accurate	Protocols
Temperature	JDRK-DH	−40–80 °C	-	±0.2 °C	Modbus-RTU
Humidity	JDRK-DH	0–100%	-	±2%	Modbus-RTU
CO_2_	JDRK-CD	0–5000 ppm	1 ppm	50 ppm	Modbus-RTU
NH_3_	JD-MQ-AM	0–200 ppm	0.2 ppm	50 ppm	Modbus-RTU
H_2_S	JD-MQ-HS	0–200 ppm	0.2 ppm	50 ppm	Modbus-RTU
Air velocity	JDRK-AV	0–30 m/s	0.1 m/s	±0.2 + 0.03 V	Modbus-RTU

**Table 2:** Optimization results of OTDBO−TCN−GRU.

Training Rounds	$\mathrm{Filter}\;F_{1}$	$\mathrm{Filter}\;F_{2}$	$\mathrm{Neuron}\;\mathrm{Numbers}\;N_{1}$	$\mathrm{Neuron}\;\mathrm{Numbers}\;N_{2}$	Optimal Fitness
1	26	41	12	27	0.01183126
2	26	41	12	27	0.01183126
…	…	…	…	…	…
7	32	127	22	128	0.00609744
8	32	127	23	128	0.00437462
9	32	127	23	128	0.00437462

**Table 3:** Contrast of the model performances.

Models	MSE	MAE	*R* ^2^
OTDBO−TCN−GRU	0.0039	0.0474	0.9871
DBO−TCN−GRU	0.0117	0.0755	0.9619
OOA	0.0163	0.0929	0.9414
GRU	0.0151	0.0924	0.9508
LSTM	0.0207	0.0980	0.9326
XGBoost	0.0376	0.1333	0.8773

**Table 4:** Effect of outdoor temperature on ammonia prediction.

Temperature	MSE	MAE	*R* ^2^
Existence outdoor temperature	0.0039	0.0474	0.9871
No outdoor temperature	0.0036	0.0463	0.9889

## Data Availability

The experimental data are original and not convenient for public disclosure. If there is a reasonable request, please contact the authors.
